# Epiphora

**Published:** 1892-04-16

**Authors:** 


### OPHTHALMOLOGY.

### EPIPHORA.

Epiphora, an overflow of tears from the eye, may result from an excessive secretion of the lachrymal fluid, or from some obstruction in the drainage apparatus, whose function it is to carry off the tears after they have passed over the eyeball, and performed their function of lubricating the conjunctival surfaces of the globe and eyelids, which in the absence of the tears would be liable to inflammation from excessive friction. The act of lachrymation is a reflex function performed by the medium of the fifth nerve; the conjunctivæ are supplied by filaments of the sensory division of the fifth nerve, which are stimulated by the friction of the eyelid, or by any foreign body entering the conjunctival sac, and reflect from the cerebral centre along the branch of the same nerve which supplies the lachrymal gland and causes the gland to secrete. Along the nasal branch of the fifth nerve may be explained in the same way the secretion of tears resulting on the stimulation of the nasal mucous membrane by snuff, volatile pungent odours, and foreign bodies. Thus we find epiphora in diseases of the eyelids and conjunctivæ, such as blepharitis, trachoma, ulcers of the cornea, &c., in diseases of the lachrymal canal, and diseases of the nasal mucous membrane; the latter two groups constitute causes of obstruction in the drainage apparatus.

The tears, after passing over the conjunctivæ, filter through or are sucked up by the puncta lachrymarum, situated at the inner angle of the eye, on the lower lid, thus into the lachrymal canaliculus, the sac, and thence down the nasal duct into the nose.

The obstruction may be at the punctum itself; this may be congenitally imperforate, or occluded, as the result of inflammation, or it may be displaced, its natural position being so that it looks backwards, resting against the eyeball, and slightly upwards. If from any cause the eyelid is relaxed, or tied down and everted (ectropion), the punctum is moved out of place, and looking upward or forward is unable to execute its sucking function on the tears; this also occurs in paralysis of the orbicularis muscle in facial paralysis.

The obstruction may be the result of stricture of the canaliculus, caused by wounds or inflammation, and is generally situated at the junction of the canaliculus with the lachrymal sac. Stricture also occurs at the junction of the lachrymal sac with the nasal duct, and where the nasal duct opens into the nose, it is sometimes in other situations the result of injury during the passage of probes, and false passages may be formed in the same way. Epiphora is produced also by nasal diseases, which interfere with the escape of the tears, diseases of the turbinated bones, polypinarium, acute or chronic rhinitis.

The treatment resolves itself into the treatment of the existing cause. When the punctum is only very slightly displaced, the use of strong astringent eyewashes may be sufficient, but when it is too much separated from the ocular conjunctiva, the duct must be entered by an artificial opening, except in cases where the displacement is due to facial paralysis. A probe-pointed knife is introduced through the punctum lachrymalis, and its sharp edge is made to cut into the canaliculus, looking backwards towards the eyeball, and slightly upwards; the knife having been introduced horizontally, the handle is now raised perpendicularly, and this completes the incision, leaving a wide opening at the inner canthus for the escape of the tears.

Stricture of the canal anywhere from the punctum to the opening in the nose is to be treated by dilatation with graduated probes; the probe is introduced horizontally after entering the punctum until it reaches the sac, its free end is then raised until it is perpendicular, when the point is made to enter the nasal duct with as little force as necessary. In these descriptions the patient is supposed to be in a sitting posture. This treatment is repeated every fourth day or so until the stricture is dilated.

Obstruction to the escape of tears down the nasal duct, if not removed, leads to chronic disease, thickening of the walls, and distension of the lachrymal sac going on to formation of dacrocysts, and lachrymal abscess.

The epiphora arising from diseases or injuries of the conjunctiva, eyelids and nasal passages, can only be cured by treatment of these lesions. In conclusion, it is necessary to remember that epiphora is not a specific disease of itself, but merely a prominent symptom of the diseases, injuries, and deformities enumerated above.

